# Frailty and outcomes in heart failure patients from high-, middle-, and low-income countries

**DOI:** 10.1093/eurheartj/ehad595

**Published:** 2023-08-28

**Authors:** Darryl P Leong, Philip Joseph, John J V McMurray, Jean Rouleau, Aldo P Maggioni, Fernando Lanas, Sanjib K Sharma, Julio Núñez, Bishav Mohan, Ahmet Celik, Jabir Abdullakutty, Okechukwu S Ogah, Lisa M Mielniczuk, Kumar Balasubramanian, Tara McCready, Alex Grinvalds, Salim Yusuf

**Affiliations:** The Population Health Research Institute, McMaster University, Hamilton Health Sciences, Hamilton General Hospital, C2-238 David Braley Building, 237 Barton St. East, Hamilton, ON L8L 2X2, Canada; Department of Medicine, McMaster University, 1280 Main Street West, Hamilton, ON, Canada; The Population Health Research Institute, McMaster University, Hamilton Health Sciences, Hamilton General Hospital, C2-238 David Braley Building, 237 Barton St. East, Hamilton, ON L8L 2X2, Canada; Department of Medicine, McMaster University, 1280 Main Street West, Hamilton, ON, Canada; British Heart Foundation Cardiovascular Research Centre, University of Glasgow, 126 University Place, Glasgow G12 8TA, UK; Department of Medicine, Université de Montréal, 2900 Edouard Montpetit Blvd, Montréal, QC H3T 1J4, Canada; ANMCO Research Center, Heart Care Foundation, Via La Marmora, 36 – 50121 Firenze, Italy; Department of Internal Medicine, Universidad de La Frontera, Temuco 4780000, Chile; B.P. Koirala Institute of Health Sciences, Buddha Road, Dharan 56700, Nepal; Servicio de Cardiología, Hospital Clínico Universitario Valencia, Avda. Blasco Ibáñez 17, 46010 Valencia, Spain; Dayanand Medical College and Hospital, Civil Lines, Tagore Nagar, Ludhiana 141001, India; Faculty of Medicine, Mersin University, 31168 Sokak, Ritim Ofis, A Blok 1 Kat, 33000 Mersin, Türkiye; Lisie Hospital, Cochin 682018, Kerala, India; Department of Medicine, University of Ibadan and University College Hospital Ibadan, PO Box 14343, Ibadan, Nigeria; University of Ottawa Heart Institute, 40 Ruskin St, Ottawa, ON K1Y 4W7, Canada; The Population Health Research Institute, McMaster University, Hamilton Health Sciences, Hamilton General Hospital, C2-238 David Braley Building, 237 Barton St. East, Hamilton, ON L8L 2X2, Canada; The Population Health Research Institute, McMaster University, Hamilton Health Sciences, Hamilton General Hospital, C2-238 David Braley Building, 237 Barton St. East, Hamilton, ON L8L 2X2, Canada; The Population Health Research Institute, McMaster University, Hamilton Health Sciences, Hamilton General Hospital, C2-238 David Braley Building, 237 Barton St. East, Hamilton, ON L8L 2X2, Canada; The Population Health Research Institute, McMaster University, Hamilton Health Sciences, Hamilton General Hospital, C2-238 David Braley Building, 237 Barton St. East, Hamilton, ON L8L 2X2, Canada; Department of Medicine, McMaster University, 1280 Main Street West, Hamilton, ON, Canada

**Keywords:** Heart failure, Frailty, Risk score, Cohort, Global health

## Abstract

**Background and Aims:**

There is little information on the incremental prognostic importance of frailty beyond conventional prognostic variables in heart failure (HF) populations from different country income levels.

**Methods:**

A total of 3429 adults with HF (age 61 ± 14 years, 33% women) from 27 high-, middle- and low-income countries were prospectively studied. Baseline frailty was evaluated by the Fried index, incorporating handgrip strength, gait speed, physical activity, unintended weight loss, and self-reported exhaustion. Mean left ventricular ejection fraction was 39 ± 14% and 26% had New York Heart Association Class III/IV symptoms. Participants were followed for a median (25th to 75th percentile) of 3.1 (2.0–4.3) years. Cox proportional hazard models for death and HF hospitalization adjusted for country income level; age; sex; education; HF aetiology; left ventricular ejection fraction; diabetes; tobacco and alcohol use; New York Heart Association functional class; HF medication use; blood pressure; and haemoglobin, sodium, and creatinine concentrations were performed. The incremental discriminatory value of frailty over and above the MAGGIC risk score was evaluated by the area under the receiver-operating characteristic curve.

**Results:**

At baseline, 18% of participants were robust, 61% pre-frail, and 21% frail. During follow-up, 565 (16%) participants died and 471 (14%) were hospitalized for HF. Respective adjusted hazard ratios (95% confidence interval) for death among the pre-frail and frail were 1.59 (1.12–2.26) and 2.92 (1.99–4.27). Respective adjusted hazard ratios (95% confidence interval) for HF hospitalization were 1.32 (0.93–1.87) and 1.97 (1.33–2.91). Findings were consistent among different country income levels and by most subgroups. Adding frailty to the MAGGIC risk score improved the discrimination of future death and HF hospitalization.

**Conclusions:**

Frailty confers substantial incremental prognostic information to prognostic variables for predicting death and HF hospitalization. The relationship between frailty and these outcomes is consistent across countries at all income levels.

## Structured Graphical Abstract

**Structured Graphical Abstract:**
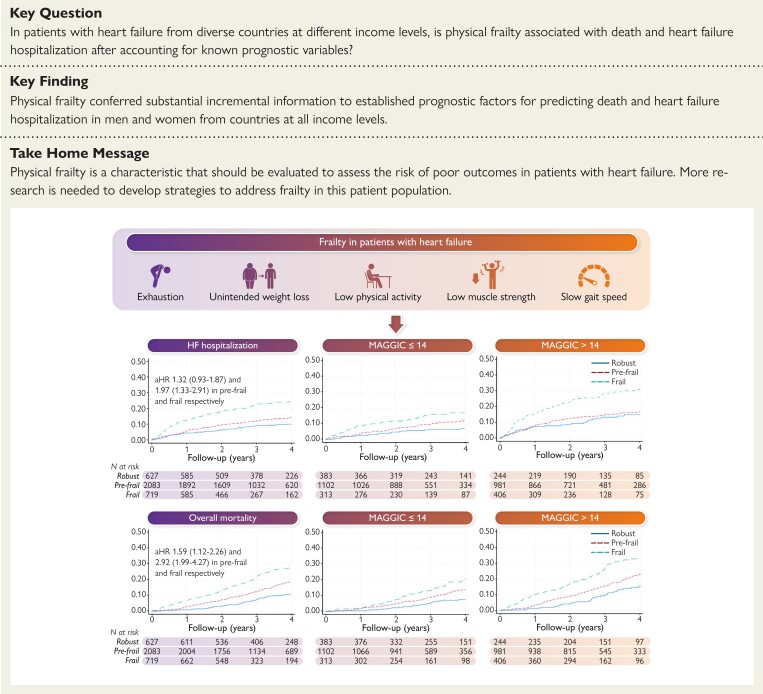
Frailty in the presence of heart failure is associated with an approximately two-fold increase in the risk of heart failure hospitalization and a three-fold increase in the risk of death as compared with robustness. aHR, adjusted hazard ratio; HF, heart failure.

**See the editorial comment for this article ‘Frailty: a new vital sign in heart failure comes of age’, by F.A. McAlister, https://doi.org/10.1093/eurheartj/ehad559.**

## Introduction

In patients with heart failure (HF), physical frailty is associated with higher risks of death and HF hospitalization. In a systematic review of HF studies, frailty was associated with a 48% higher risk of death [hazard ratio (HR) 1.48, 95% confidence interval (CI) 1.31–1.65] and a 40% higher risk of HF hospitalization (HR 1.40, 95% CI 1.27–1.54) as compared with not being frail.^1^ However, the independent and incremental prognostic value of frailty over and above established conventional HF variables is uncertain. Also, most HF occurs in countries outside North America and Europe, but there are few data on the effects of frailty on HF outcomes from these regions. In a systematic review of frailty in HF, of the 29 studies and 18 757 participants identified, only three studies with 236 patients were from countries outside North America, Australasia, and Europe.^1^ Therefore, parts of the world accounting for most HF cases^2^ are only represented by 1% of the data on frailty in HF.

While frailty is associated with worse outcomes among patients with HF, it is not known whether its effects vary among different subgroups of patients. Therefore, the objectives of this study were to (i) evaluate the relationship between frailty and the outcomes of death and HF hospitalization in HF patients from countries at all income levels and whether the strength of the associations varies among regions; (ii) describe the relationship between frailty and the outcomes of death and HF hospitalization in different modifiable subgroups; and (iii) evaluate the independent and incremental prognostic value of frailty compared with other commonly used HF prognostic indices.

## Methods

### Patient population

This study is an analysis of the Global Congestive Heart Failure (G-CHF) registry, whose design has previously been described.^3,4^ The G-CHF is a multi-centre prospective cohort study of patients with a clinical diagnosis of HF conducted in 40 high (HIC)-, upper middle (UMIC)-, lower middle (LMIC), and low (LIC)-income countries from eight geographic regions. Study sites were selected based on prior experience collaborating with the coordinating centre, the Population Health Research Institute, Canada. Sites were guided to recruit approximately two-thirds of participants from ambulatory care settings and one-third from hospital inpatients. Outpatients and inpatients with either newly diagnosed or previously diagnosed HF of any aetiology were eligible. Patients < 18 years of age were excluded.

A subset of participating centres in G-CHF elected to recruit patients into a substudy based on their capacity to collect the extra data required. Individuals at these sites who provided consent for more detailed characterization as part of the G-CHF substudy underwent collection of data on frailty at baseline as subsequently described. The present analysis is based on these individuals, who each provided informed consent to this research. The study was approved by the relevant institutional review boards.

### Baseline characteristics

At the baseline study visit, information on participant demographics, HF aetiology, New York Heart Association (NYHA) functional class, co-morbidities, alcohol and tobacco use, HF therapies, serum sodium, and haemoglobin concentrations was collected. Anaemia was considered present if the haemoglobin was <130 g/L in men or <120 g/L in women. We collected left ventricular ejection fraction (LVEF) as measured at the site. Left ventricular ejection fraction of <40% was considered reduced. We documented B-type natriuretic peptide (BNP) or N-terminal pro-BNP (NT-proBNP) concentrations when these were measured for clinical purposes. We considered natriuretic peptide levels to be elevated if BNP was >100 pg/mL or NT-proBNP was >300 pg/mL.^5^ We calculated each participant’s MAGGIC risk score as previously described.^6^

Frailty was assessed in a standardized manner using the approach described by Fried *et al.*^7^ This index is the most commonly used tool to assess physical frailty in the general population and in HF cohort studies.^1^ Handgrip strength was measured twice in each hand using a handgrip dynamometer, and measurements were averaged. Low handgrip strength was considered present in women if ≤17 kg in those with body mass index (BMI) ≤ 23 kg/m^2^; ≤17.3 kg in those with BMI >23 and ≤26 kg/m^2^; ≤18 kg in those with BMI >26 and ≤29 kg/m^2^; or ≤21 kg in those with BMI > 29 kg/m^2^. In men, low handgrip strength was considered present if ≤29 kg in those with BMI ≤ 24 kg/m^2^; ≤30 kg in those with BMI >24 and ≤28 kg/m^2^; or ≤32 kg in those with BMI > 28 kg/m^2^. Gait speed was evaluated using the timed get-up-and-go test, in which the time needed for the participant to rise from a chair, walk 3 m, return to the chair, and sit down was recorded using a stopwatch. Gait speed was considered slow if the test required >9 s in those aged <70 years; >10.2 s in those aged ≥70 and <80 years; and >12.7 s in those aged ≥80 years.^8^ Physical activity was measured using the International Physical Activity Questionnaire^9^ and was considered low if less than the lowest sex-specific quintile for the cohort.^7^ Unanticipated weight loss was considered present if the participant reported involuntary weight loss of >3 kg in the preceding 6 months. Exhaustion was considered present if the participant reported feeling that everything was an effort ≥3 days during the preceding week.

Participants were classified as frail if they exhibited three or more of low handgrip strength; slow gait; low physical activity; unanticipated weight loss; and exhaustion. Participants were classified as pre-frail in the presence of one or two of these characteristics and robust if they had none of these characteristics.

In addition to the Fried index, we also evaluated frailty by developing a cumulative deficit model.^10^ Each of 22 deficits listed in the *Supplementary Materials* was given a value of 0 if absent vs. 1 if present. Deficits present for each participant were summed, yielding a score with a maximum possible value of 22, and then each participant’s score was divided by 22 to calculate a deficit index with a minimum value of 0 and a maximum value of 1. We defined pre-frailty by cumulative deficit index as a cumulative deficit index of >0.1 and ≤0.21 and frailty as a cumulative deficit index >0.21 as previously described in a large cohort of participants with or at high risk of cardiovascular disease.^10^

### Follow-up

Participants were contacted every 6 months to determine vital status and to identify hospitalizations. Median and maximum follow-up times were 3.1 and 6.1 years, respectively. During this time, 89 (2.6%) of participants were lost to follow-up.

### Statistical analysis

We performed Cox proportional hazards models with participant ethnicity as a random effect to account for clustering of characteristics within ethnicities.^11^ Physical frailty was modelled as the exposure of interest and adjustment was made for characteristics that have been shown to have prognostic value in cardiovascular disease: age; sex; country income level; education; HF aetiology; LVEF; NYHA functional class; diabetes; and the use of alcohol, tobacco, angiotensin-converting enzyme inhibitors/angiotensin receptor blockers (ACE-I/ARB) and beta-blockers; BMI, baseline creatinine, sodium, and systolic blood pressure. BMI, creatinine, sodium levels, and blood pressure were modelled as continuous untransformed variables. We repeated these models with interaction terms between physical frailty and respectively: age (≤65 years vs. >65 years), sex, education, alcohol, tobacco, diabetes, anaemia, creatinine (stratified by the sample median of 93 µmol/L), NYHA functional class, LVEF (stratified by ≥40% vs. <40%), ACE-I/ARB and beta-blocker use, MAGGIC risk score (stratified by the cohort median value of 14), and country income level.

To determine whether the measurement of frailty confers incremental prognostic value to accepted HF prognostic factors, we compared rates and models for death and HF hospitalization using the MAGGIC risk score^6^ alone vs. the MAGGIC risk score combined with the frailty score. This evaluation was performed by (i) calculating age- and sex-standardized event rates stratified by levels of MAGGIC risk score and frailty; (ii) comparing Akaike’s information criteria (AICs) for Cox models containing the MAGGIC score alone vs. the MAGGIC score and frailty; (iii) comparing areas under the receiver-operating characteristic curve when adding the frailty score to the MAGGIC score^12^; and (iv) calculating the net re-classification improvement^13^ when adding the frailty score to the MAGGIC score. For the calculation of net re-classification improvement, we empirically considered a mortality or HF hospitalization rate of 15% during the 3.1 years of follow-up to be high as the crude mortality and HF hospitalization rates were 16% and 14% during this time.

To compare frailty as measured using Fried’s frailty phenotype vs. the cumulative deficit index, we performed Cox proportional hazards models containing both the frailty phenotype and cumulative deficit index. These models were adjusted for age, country income level, and sex.

## Results

### Baseline characteristics

The baseline characteristics of participants are described in *Table 1*. Among 3429 individuals, 627 (18%) were robust, 2083 (61%) were pre-frail, and 719 (21%) were frail. Frailty was associated with older age. More women (24%) than men (20%) were frail. Fewer individuals in HIC (14%) were frail, as compared with UMIC (29%), LMIC (22%), or LIC (23%). There was an inverse association between education and frailty: 30% of those with primary school education were frail vs. 18% of those with secondary education and 14% of those with education beyond secondary school. Current alcohol use (14%) and tobacco (14%) use were associated with lower rates of frailty than former alcohol (21%) or tobacco (19%) use or never having used alcohol (25%) or tobacco (23%). Diabetes was associated with frailty: 25% of those with diabetes were frail as compared with 20% of those without diabetes. Frailty was strongly associated with NYHA functional class, with prevalence rates of 10%, 19%, 30%, and 42% among those with Class I, II, III, and IV symptoms, respectively (*P* < .0001). There was no association between frailty and HF duration or LVEF. There was an inverse association between haemoglobin and frailty, with haemoglobin levels 139 ± 19 g/L, 134 ± 19 g/L, and 129 ± 21 g/L in robust, pre-frail, and frail participants, respectively (*P* < .0001), and frailer individuals had higher creatinine levels. Frailty was not related to the use of ACE-I/ARB or beta-blockers, but it was positively related to the MAGGIC score.

**Table 1:** Characteristics of patients based on frailty phenotype

Characteristic	Overall *N* = 3429	Robust *n* = 627	Pre-frail *n* = 2083	Frail *n* = 719	*P*-value
Age, years	61.2 ± 14.3	60.4 ± 12.7	60.8 ± 14.3	63.1 ± 14.3	.0002
Sex					<.0001
Female	1146	160 (26)	713 (34)	273 (38)	
Male	2283	467 (74)	1370 (66)	446 (62)	
Country income					<.0001
High	1311	379 (60)	747 (36)	185 (26)	
Upper middle	805	67 (11)	501 (24)	237 (33)	
Lower middle	926	112 (18)	606 (29)	208 (29)	
Low	387	69 (11)	229 (11)	89 (12)	
Education					<.0001
Primary	1236	150 (24)	720 (35)	366 (51)	
Secondary	1201	236 (38)	750 (36)	213 (30)	
>Secondary	989	240 (38)	612 (29)	136 (19)	
Tobacco					<.0001
Never	1841	275 (44)	1135 (55)	431 (60)	
Former	1262	288 (46)	730 (35)	244 (34)	
Current	325	63 (10)	218 (10)	44 (6)	
Alcohol					<.0001
Never	1665	212 (34)	1036 (50)	417 (58)	
Former	784	133 (21)	484 (23)	167 (23)	
Current	979	281 (45)	563 (27)	135 (19)	
Diabetes	925	136 (22)	561 (27)	228 (32)	<.0001
HF aetiology					<.0001
Ischaemia	1317	242 (39)	821 (39)	254 (35)	
Hypertension	611	80 (13)	370 (18)	161 (23)	
Idiopathic	622	139 (22)	352 (17)	131 (18)	
Valve/rheumatic	255	45 (7)	152 (7)	58 (8)	
Other	624	121 (19)	388 (19)	115 (16)	
NYHA functional class					<.0001
I	556	171 (28)	328 (16)	57 (8)	
II	1975	363 (58)	1234 (59)	376 (52)	
III/IV	891	89 (14)	517 (25)	285 (40)	
Hospitalized within the previous 2 years	1686	308 (49)	1011 (49)	367 (51)	.51
Mean HF duration, years	4.0 ± 1.4	4.0 ± 1.4	3.9 ± 1.4	4.0 ± 1.4	.27
LVEF, %	39 ± 14	40 ± 14	39 ± 14	39 ± 14	.72
Body mass index, kg/m^2^	27.6 ± 5.9	27.9 ± 5.2	27.5 ± 5.8	27.7 ± 6.6	.38
Systolic blood pressure, mmHg	123 ± 20	124 ± 19	123 ± 20	122 ± 20	.16
Serum sodium, mmol/L	139 ± 4	140 ± 4	139 ± 4	139 ± 4	.16
Serum creatinine, µmol/L	108 ± 74	104 ± 81	107 ± 67	115 ± 87	.038
Haemoglobin, g/L	134 ± 20	139 ± 19	134 ± 19	129 ± 21	<.0001
ACE-I/ARB use	2520	480 (77)	1527 (73)	513 (71)	.093
Beta-blocker use	2888	532 (85)	1756 (84)	600 (83)	.77
MRA use	2046	352 (56)	1260 (60)	434 (60)	.14
Elevated natriuretic peptide levels^a^	905 (81)	187 (68)	538 (84)	180 (90)	<.0001
MAGGIC score	15.5 ± 7.2	14.0 ± 6.4	15.3 ± 7.1	17.1 ± 7.7	<.0001

Numbers in parentheses represent column percentages.

ACE-I/ARB, angiotensin-converting enzyme inhibitors/angiotensin receptor blockers; HF, heart failure; LVEF, left ventricular ejection fraction; MRA, mineralocorticoid receptor antagonist.

Clinically measured natriuretic peptide levels were available in 1113 participants.

### The relationship between frailty and mortality

During a median (25th to 75th percentile) of 3.1 (2.0–4.3) years of follow-up, 565 (16%) participants died. Age- and sex-standardized mortality rates stratified by MAGGIC risk score quintile are displayed in *Table 2*. In further analyses, we calculated age- and sex-standardized mortality rates stratified by the median MAGGIC risk score (see Supplementary data online, *Table S1*). These analyses show that for any given MAGGIC risk score, mortality rates are higher in frail individuals.

**Table 2:** Age- and sex-standardized mortality and heart failure hospitalization rates stratified by MAGGIC score quintile, both overall and in frail individuals

MAGGIC score quintile	Age- and sex-standardized mortality rate	Age- and sex-standardized mortality rate in frail individuals	Age- and sex-standardized HF hospitalization rate	Age- and sex-standardized HF hospitalization rate in frail individuals
**1**	2.5 (2.0–3.1)	4.2 (2.1–6.3)	2.4 (1.8–2.9)	4.7 (3.5–6.0)
**2**	4.7 (3.9–5.6)	5.5 (3.4–7.5)	4.2 (3.4–5.0)	9.4 (6.7–12.0)
**3**	5.8 (4.6–7.1)	6.4 (3.8–9.0)	4.6 (3.5–5.7)	3.4 (0.9–5.8)
**4**	5.3 (4.3–6.3)	6.8 (4.5–9.1)	5.6 (4.6–6.6)	7.4 (4.6–10.2)
**5**	11.0 (8.5–13.5)	13.2 (8.7–17.8)	8.0 (5.8–10.2)	11.6 (6.9–16.3)

HF, heart failure.

**Figure 1:**
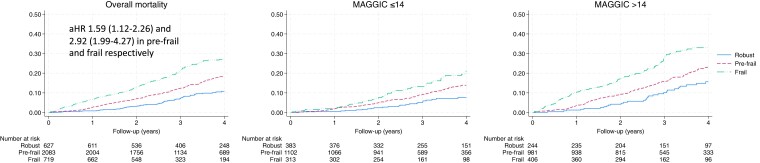
Kaplan–Meier curves demonstrating the relationship between frailty and time to death, both overall and stratified by the cohort’s median MAGGIC risk score. Interaction *P* value between frailty and MAGGIC score was *P* = .051.

As compared with robust participants, pre-frail and frail individuals had respective adjusted HRs (95% CI) for death of 1.59 (1.12–2.26) and 2.92 (1.99–4.27) as compared with robust individuals (*Figure 1*). In sensitivity analyses, we described the relationship between the individual components of the frailty index and mortality, as well as different combinations of these individual components. In general, the full frailty index identified more vulnerable individuals and was associated with higher hazards than individual or combinations of two to three frailty characteristics (see Supplementary data online, *Table S2*).

Natriuretic peptide levels were available in 1113 participants. In a sensitivity analysis, a Cox model including only frailty and natriuretic peptide levels demonstrated that pre-frailty and frailty were associated with a higher risk of death independently of natriuretic peptides. The respective HRs (95% CI) among the pre-frail and frail were 2.00 (1.25–3.21) and 3.23 (1.90–5.51). However, after adjusting for all covariates, the model would not converge, so fully adjusted estimates for death are not available.

The relationship between frailty and the risk of death was consistent among men and women (*Table 3* and Supplementary data online, *Figure S1*). In addition, there was no evidence of heterogeneity in the relationship between frailty and mortality across country income levels (*Table 3*), nor was there evidence of heterogeneity in the relationship between frailty and mortality in most other subgroups, although there was modest heterogeneity in the relationship between frailty and mortality across education levels (interaction *P* = .016). Further detail is provided in Supplementary data online, *Table S3* and *Figure S1*.

**Table 3:** Cox models for death and heart failure hospitalization stratified by sex and country income level

Subgroups	Death	Interaction *P* value	Heart failure hospitalization	Interaction *P* value
Sex		.25		.44
Male				
Robust	1		1	
Pre-frail	1.62 (1.08–2.43)		1.43 (0.95–2.15)	
Frail	3.21 (2.07–4.99)		2.31 (1.45–3.67)	
Female				
Robust	1		1	
Pre-frail	1.30 (0.65–2.62)		1.05 (0.53–2.08)	
Frail	1.99 (0.94–4.23)		1.44 (0.68–3.09)	
Upper middle or high-income countries		.29		.36
Robust	1		1	
Pre-frail	1.69 (1.06–2.68)		1.53 (1.00–2.35)	
Frail	2.44 (1.44–4.13)		2.44 (1.49–3.99)	
Low-middle or low-income countries				
Robust	1		1	
Pre-frail	1.24 (0.72–2.12)		0.98 (0.54–1.80)	
Frail	2.61 (1.48–4.60)		1.33 (0.68–2.60)	

Interaction *P* values refer to the interaction between frailty and the respective subgroups.

### Frailty and heart failure hospitalization

During follow-up, 471 (14%) participants were hospitalized for HF. Age- and sex-standardized HF hospitalization rates by strata of MAGGIC risk score both overall and in frail individuals within each stratum are shown in *Table 2* and Supplementary data online, *Table S1*. These analyses show that for any given MAGGIC risk score, HF hospitalization rates are higher in frail individuals.

**Figure 2:**
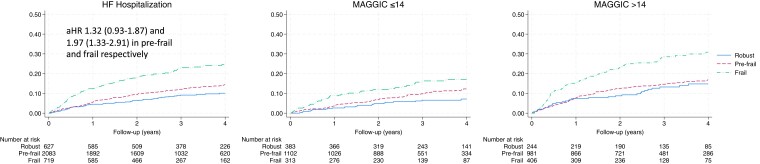
Kaplan–Meier curves demonstrating the relationship between frailty and time to heart failure hospitalization, both overall and stratified by the cohort’s median MAGGIC risk score. Interaction *P* value between frailty and MAGGIC score was *P* = .22.

As compared with robust participants, the adjusted HRs (95% CI) for HF hospitalizations among pre-frail and frail individuals were 1.32 (0.93–1.87) and 1.97 (1.33–2.91) (*Figure 2*). Therefore, as observed with mortality, the relationship between frailty and HF hospitalization is independent of important prognostic factors. In sensitivity analyses, we described the relationship between the individual components of the frailty index and HF hospitalization, as well as different combinations of these individual components. In general, the full frailty index identified more vulnerable individuals and was associated with higher hazards than individual or combinations of two to three characteristics (see Supplementary data online, *Table S2*).

In a sensitivity analysis, a Cox model including only frailty and natriuretic peptide levels demonstrated that pre-frailty and frailty were associated with an increased risk of death independently of natriuretic peptides. The respective HRs (95% CI) among the pre-frail and frail were 1.80 (1.12–2.89) and 3.92 (2.33–6.58). After adjusting for all covariates, the respective HRs (95% CI) among the pre-frail and frail were 1.50 (0.90–2.51) and 2.96 (1.62–5.41).

The relationship between frailty and the risk of HF hospitalization was consistent among men and women (*Table 3* and Supplementary data online, *Figure S1*). There was modest evidence of heterogeneity in the relationship between frailty and HF hospitalization across country income levels (interaction *P* = .036). In HIC and UMIC, the risk of HF hospitalization was elevated in pre-frail and frail individuals; however, the likelihood of being hospitalized for HF was attenuated in pre-frail and frail individuals from LMIC and LIC.

Other subgroup analyses are presented in Supplementary data online, *Table S3*. There was no evidence of heterogeneity among these subgroups in the relationship between frailty and HF hospitalization with the possible exception of diabetes.

### The incremental prognostic value of frailty

**Figure 3:**
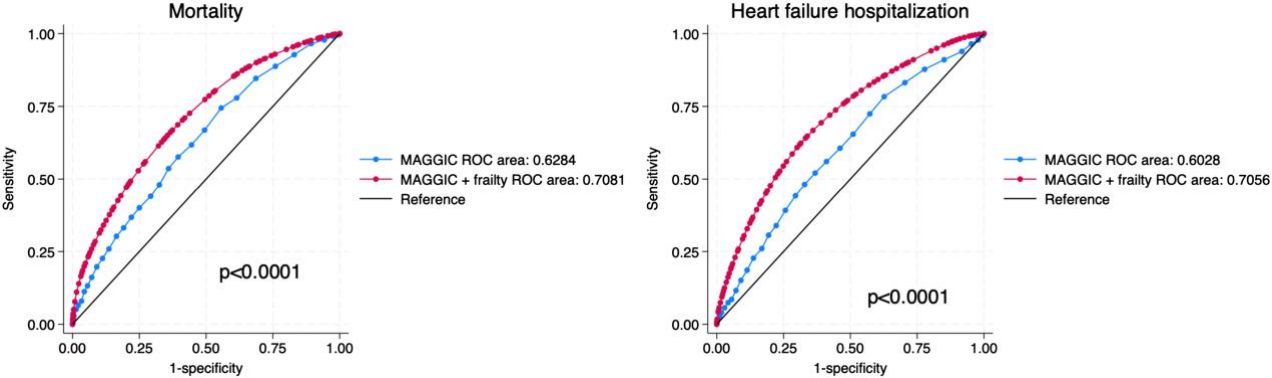
Receiver-operating characteristic curves for mortality and heart failure hospitalization. *P* values refer to the comparison of the MAGGIC score alone with the combined MAGGIC score and frailty. ROC, receiver-operating characteristic curve.

We compared models for overall mortality with the MAGGIC risk score as the only covariate vs. the MAGGIC risk score and the frailty score as covariates. In this latter model, both the MAGGIC score and the frailty score were independently associated with mortality (all *P* < .001). The AIC was lower in the model containing both the MAGGIC score and the frailty score (AIC = 8414) than the model containing the MAGGIC score alone (AIC = 8475), indicating that the model containing both scores fit the data better than the model containing the MAGGIC score alone. Areas under the receiver-operating characteristic curve were 0.62 for the MAGGIC score alone vs. 0.71 for the MAGGIC score combined with the frailty score (*P* < .0001), indicating that measuring both scores better identified patients more likely to die than the MAGGIC score alone (*Figure 3*). The net re-classification improvement after adding frailty to the model containing the MAGGIC score was 0.19 (*P* < .0001), indicating that the additional measurement of frailty improved the classification of HF patients’ risk of death in 19% of cases.

We also compared models for overall HF hospitalization with the MAGGIC risk score as the only covariate vs. the MAGGIC risk score and the frailty score as covariates. In the model containing the two risk scores, both the MAGGIC score and the frailty score were independently associated with mortality (all *P* < .0001). The AIC was lower in the model containing both the MAGGIC score and the frailty score (AIC = 7283) than the model containing the MAGGIC score alone (AIC = 7334), indicating that the model containing both scores fit the data better than the model containing the MAGGIC score alone. Areas under the receiver-operating characteristic curve were 0.60 for the MAGGIC score alone vs. 0.71 for the MAGGIC score combined with the frailty score (*P* < .0001), indicating that measuring both scores better identified patients more likely to be hospitalized for HF than the MAGGIC score alone (*Figure 3*). The net re-classification improvement after adding frailty to the model containing the MAGGIC score was 0.13 (*P* < .0001), indicating that the additional measurement of frailty improved the classification of HF patients’ risk of HF hospitalization by 13%.

Using the cumulative deficit index, 839 participants (25%) were considered robust, 1078 (31%) pre-frail, and 1512 (44%) frail. After adjustment for age, sex, and country income level, both pre-frailty and frailty as measured by both the Fried phenotype and the cumulative deficit indices were independently associated with the risk of death or HF hospitalization. However, the risk of death or HF hospitalization was higher among pre-frail and frail individuals as identified by the Fried frailty phenotype as compared with the cumulative deficit model (*Table 4*).

**Table 4:** Cox models for death and heart failure hospitalization containing both the frailty phenotype and the cumulative deficit index

Outcome	Frailty level	Frailty phenotype	Frailty by cumulative deficits
**Mortality**	Robust	1	1
	Pre-frail	1.73 (1.29–2.30)	1.28 (1.01–1.63)
	Frail	2.78 (2.02–3.82)	1.39 (1.08–1.79)
**Heart failure hospitalization**	Robust	1	1
	Pre-frail	1.47 (1.09–1.99)	1.24 (0.92–1.66)
	Frail	2.57 (1.84–3.59)	2.16 (1.61–2.90)

Adjustment was made for age, sex, and country income level.

## Discussion

The main findings from this analysis are as follows: (i) there is a strong, progressive association between pre-frailty and frailty vs. the risk of death or HF hospitalization; (ii) the relationship between frailty and death or HF hospitalization is independent of conventional prognostic variables; and (iii) the association between frailty and death or HF hospitalization is consistent across many different modifiable clinical characteristics, suggesting that addressing these characteristics is unlikely to mitigate the impact of frailty on adverse clinical outcomes (*Structured Graphical Abstract*).

### The relationship between frailty and adverse outcomes in patients with heart failure

Our findings are consistent with those of the FRAIL-HF study.^14^ In this prospective cohort study of 450 patients hospitalized with HF conducted at a single Spanish hospital, frailty was evaluated in the same way as in our study. Frailty was associated with a two-fold increase in the risk of 1-year re-admission and of 1-year mortality. A multi-centre Spanish study of 497 participants using the same frailty assessment tool demonstrated similar results.^15^ A systematic review that included studies examining the relationship between frailty and mortality or hospitalizations in HF patients identified 20 relevant studies.^16^ These studies used heterogeneous approaches to evaluate frailty. Collectively, they found that frailty was associated with HR (95% CI) for mortality of 1.59 (1.39–1.82) and for hospitalization of 1.31 (1.21–1.42). The weaker association between frailty and adverse outcomes as compared with our study may be related to the tools that were used to evaluate frailty.

Our study is several times larger than any previous prospective study on frailty in HF patients, and it extends the existing research by demonstrating that the prognostic importance of frailty is consistent in 27 countries at all income levels and is not influenced by smoking, alcohol, systolic left ventricular function, anaemia, or the use of ACE-I/ARB or beta-blockers. Natriuretic peptide levels are strong predictors of death and HF hospitalization in patients with HF.^17^ In a subgroup of patients, we found that frailty is associated with the risk of death or HF hospitalization independently of natriuretic peptide levels, highlighting the prognostic importance of frailty in this population.

To the extent that more severe HF may lead to the development of frailty, it is unsurprising that frailer individuals are at higher risk of HF hospitalization. However, we found that frailty is associated with HF hospitalization independent of characteristics known to predict HF hospitalization. This raises the possibility that frailty may predispose to the need for HF hospitalization through mechanisms other than HF in and of itself. By definition, frailty is the increased vulnerability to adverse outcomes when exposed to a physiologic stress. Frailty may lead to less functional reserve to be able to compensate when faced with stressors such as inter-current illness.

### Strategies to mitigate the possible effects of frailty in heart failure

We were unable to find a subgroup of HF patients in whom the associations between frailty and adverse outcomes were attenuated. Therefore, we remain uncertain whether addressing the modifiable characteristics studied will decrease the risk of death or HF hospitalization more so in frail individuals over and above their expected benefits in the entire HF population.

Strategies to address aspects of the frailty phenotype have been evaluated. Resistance training is effective at increasing muscle strength (an important part of the frailty phenotype) in patients with HF.^18^ Whether this translates to reduced mortality or HF hospitalization is uncertain. A systematic review of randomized trials of cardiac rehabilitation in older patients with HF demonstrated that this intervention improved 6-min walk distance and decreased hospitalization, with odds ratio (95% CI) 0.32 (0.21–0.49).^19^ Another systematic review confirmed that exercise-based cardiac rehabilitation decreases the risk of HF hospitalization.^20^ In this systematic review, there were 33 trials that included mortality as an endpoint (666 deaths in 5441 participants), but almost all (99%) were from Europe and North America. Mortality was not decreased, with relative risk (95% CI) 0.89 (0.66–1.21). The median duration of the exercise intervention was 6 months. Whether more prolonged exercise-based cardiac rehabilitation will reduce mortality by ameliorating frailty merits further investigation.

The graded association between pre-frailty and frailty with death and HF hospitalization suggests that intervening in pre-frail individuals to prevent them from becoming frail might be one strategy to decrease the impact of frailty on adverse clinical outcomes. If the Fried model of frailty used in this study is considered a continuum in which individuals acquire an increasing number of frailty characteristics over time, identifying the development of these markers of frailty early may enable the implementation of strategies to prevent frailty from developing. This is especially important in patients with HF because the physical characteristics of frailty may often be attributed to the HF itself or to a natural consequence of ageing, whereas our data suggest that frailty characteristics of themselves confer incremental prognostic information to conventional prognostic variables in HF, including age.

Apart from considering strategies to prevent frailty from developing or even reversing frailty after it develops, it is important to ensure that frail patients with HF receive appropriate guideline-directed therapies for HF. Butt *et al.*^21,22^ showed that using a cumulative deficit index to characterize frailty, dapagliflozin led to less worsening of HF or cardiovascular death and improved health-related quality of life than placebo irrespective of the participant’s level of frailty. Butt *et al.*^23^ also reported that individuals with HF with preserved ejection fraction with higher frailty scores according to a cumulative deficit model may experience fewer HF hospitalizations or cardiovascular death than those with lower frailty scores. These analyses highlight the importance of appropriate medical therapy in frail individuals with HF.

### Limitations

Our study cohort represents a relatively stable HF population because participants had to be able to complete the physical measurements needed to assess frailty. Therefore, this cohort may not be completely representative of more acutely unwell patients with HF, in whom the risk of death or HF hospitalization may be higher.

## Conclusions

Increasing levels of physical frailty are associated with progressively higher risk of both HF hospitalization and death in countries at all income levels. These relationships are independent of conventional prognostic variables, to which it adds predictive value. The association between frailty and death or HF hospitalization is consistent across many different modifiable clinical characteristics. These findings suggest that frailty may be an important therapeutic target because addressing frailty might represent a new strategy, distinct from established effective treatments, to improve outcomes in individuals with HF.

## Supplementary data

Supplementary data are available at *European Heart Journal* online.

## Supplementary Material

ehad595_Supplementary_Data

## Data Availability

Individual-level data are not available to the public as per the study policy. Summary data and the statistical code can be made available on reasonable request.
