# Supplementary material for: Frailty and outcomes in heart failure patients from high-, middle-, and low-income countries
Source: Eur Heart J. 2023 Aug 28;44(42):4435–44. doi: 10.1093/eurheartj/ehad595 (PMC10635666; doi:10.1093/eurheartj/ehad595)
Supplement: ehad595_Supplementary_Data [file ehad595_supplementary_data.docx]

**Supplementary Material**

**Supplementary Table 1. Age- and sex-standardized mortality and heart failure hospitalization rates per 100 person-years**

|  | Robust | Pre-frail | Frail |
| --- | --- | --- | --- |
| Mortality | | | |
| MAGGIC score ≤14 | 1.9 (1.1-2.7)  N=383 | 3.6 (3.0-4.3)  N=1102 | 5.0 (3.5-6.5)  N=313 |
| MAGGIC score >14 | 3.8 (2.5-5.2)  N=244 | 5.8 (5.0-6.6)  N=981 | 8.7 (7.1-10.4)  N=406 |
| Heart failure hospitalization | | | |
| MAGGIC score ≤14 | 2.0 (1.1-2.9)  N=383 | 3.1 (2.5-3.7)  N=1102 | 4.6 (3.2-6.1)  N=313 |
| MAGGIC score >14 | 3.8 (2.5-5.1)  N=244 | 4.7 (4.0-5.4)  N=981 | 9.4 (7.6-11.2)  N=406 |

**Supplementary Table 2. Hazard ratios (HR) and 95% confidence intervals (CI) for death and heart failure hospitalization observed with each component and with combinations of individual components of the full Fried frailty index.**

| Exposure | Proportion of cohort affected | Adjusted HR (95% CI) for death | Adjusted HR (95% CI) for heart failure hospitalization |
| --- | --- | --- | --- |
| Unintended weight loss | 14% | 1.74 (1.32-2.27) | 1.18 (0.86-1.63) |
| Exhaustion | 21% | 1.28 (0.99-1.65) | 1.76 (1.37-2.28) |
| Low physical activity | 30% | 1.57 (1.27-1.95) | 1.27 (1.00-1.62) |
| Low handgrip strength | 38% | 1.31 (1.04-1.64) | 1.08 (0.84-1.38) |
| Slow gait speed | 55% | 1.53 (1.22-1.93) | 1.46 (1.14-1.87) |
| Either unintended weight loss or exhaustion | 30% | 1.47 (1.17-1.84) | 1.69 (1.33-2.14) |
| Either unintended weight loss or exhaustion or low physical activity | 50% | 1.57 (1.26-1.95) | 1.81 (1.42-2.29) |
| Either unintended weight loss or exhaustion or low physical activity or slow gait speed | 75% | 2.03 (1.51-2.71) | 1.70 (1.26-2.30) |
| Pre-frail  Frail | 61%  21% | 1.59 (1.12-2.26)  2.92 (1.99-4.27) | 1.32 (0.93-1.87)  1.97 (1.33-2.91) |
| Frail or pre-frail | 82% | 1.81 (1.29-2.55) | 1.43 (1.02-2.01) |

**Supplementary Table 3**. **Subgroup analyses.** NC = model unable to converge

| Outcome | Exposure | Hazard ratio (95% confidence interval) | p-value for the interaction between the exposure and frailty |
| --- | --- | --- | --- |
| Mortality | Age  ≤65 years   - Robust - Pre-frail - Frail   >65 years   - Robust - Pre-frail - Frail | 1  1.41 (0.89-2.23)  2.48 (1.50-4.11)  1  1.73 (1.00-2.99)  3.37 (1.86-6.12) | 0.68 |
|  | Tobacco use  Current  Former   - Robust - Pre-frail - Frail   Never   - Robust - Pre-frail - Frail | NC  1  1.29 (0.76-2.17)  3.59 (2.02-6.38)  1  1.86 (1.08-3.21)  3.03 (1.69-5.44) | 0.57 |
|  | Alcohol use  Current   - Robust - Pre-frail - Frail   Former  Never   - Robust - Pre-frail - Frail | 1  1.93 (0.99-3.73)  3.13 (1.42-6.88)  NC  1  1.55 (0.86-2.79)  3.32 (1.79-6.28) | 0.68 |
|  | New York Heart Association functional class  I/II   - Robust - Pre-frail - Frail   III/IV | 1  1.73 (1.16-2.59)  2.77 (1.75-4.39)  NC | 0.39 |
|  | Left ventricular ejection fraction  ≥40%   - Robust - Pre-frail - Frail   <40%   - Robust - Pre-frail - Frail | 1  1.65 (0.91-3.01)  2.23 (1.13-4.40)  1  1.25 (0.78-2.01)  2.91 (1.75-4.85) | 0.20 |
|  | No diabetes   - Robust - Pre-frail - Frail   Diabetes | 1  1.53 (1.00-2.36)  2.50 (1.55-4.03)  NC | 0.74 |
|  | No anemia   - Robust - Pre-frail - Frail   Anemia   - Robust - Pre-frail - Frail | 1  1.50 (1.00-2.25)  2.87 (1.80-4.58)  1  1.79 (0.88-3.64)  3.18 (1.53-6.63) | 0.85 |
|  | Serum creatinine below the median for the cohort (93umol/L)   - Robust - Pre-frail - Frail   Serum creatinine above the median for the cohort   - Robust - Pre-frail - Frail | 1  1.00 (0.58-1.73)  2.03 (1.11-3.71)  1  1.96 (1.24-3.09)  3.53 (2.14-5.81) | 0.31 |
|  | Not taking an ACE-I or angiotensin receptor blocker  Taking an ACE-I or angiotensin receptor blocker   - Robust - Pre-frail - Frail | NC  1  1.91 (1.21-3.01)  3.70 (2.24-6.12) | 0.16 |
|  | Not taking a beta-blocker   - Robust - Pre-frail - Frail   Taking a beta-blocker   - Robust - Pre-frail - Frail | 1  1.62 (0.64-4.05)  2.53 (0.93-6.91)  1  1.48 (1.01-2.17)  2.85 (1.88-4.32) | 0.83 |
|  | Body mass index ≤30kg/m^2^   - Robust - Pre-frail - Frail   Body mass index >30kg/m^2^   - Robust - Pre-frail - Frail | 1  1.62 (1.05-2.49)  3.26 (2.05-5.18)  1  1.50 (0.81-2.79)  1.79 (0.88-3.65) | 0.23 |
|  | MAGGIC risk score ≤14  MAGGIC risk score >14   - Robust - Pre-frail - Frail | NC  1  1.29 (0.83-2.01)  2.81 (1.74-4.53) | 0.065 |
| Heart failure hospitalization | Age  ≤65 years   - Robust - Pre-frail - Frail   >65 years   - Robust - Pre-frail - Frail | 1  1.20 (0.77-1.88)  1.77 (1.07-2.95)  1  1.46 (0.84-2.57)  2.39 (1.27-4.49) | 0.98 |
|  | Primary or secondary education   - Robust - Pre-frail - Frail   Post-secondary education   - Robust - Pre-frail - Frail | 1  1.57 (1.01-2.44)  2.31 (1.42-3.75)  1  1.10 (0.61-1.99)  1.86 (0.87-4.00) | 0.45 |
|  | Tobacco use  Current   - Robust - Pre-frail - Frail   Former   - Robust - Pre-frail - Frail   Never   - Robust - Pre-frail - Frail | 1  1.02 (0.29-3.61)  1.27 (0.24-6.63)  1  1.47 (0.89-2.40)  2.07 (1.15-3.73)  1  1.38 (0.77-2.45)  2.01 (1.07-3.77) | 0.82 |
|  | Alcohol use  Current   - Robust - Pre-frail - Frail   Former   - Robust - Pre-frail - Frail   Never   - Robust - Pre-frail - Frail | 1  1.28 (0.72-2.29)  1.38 (0.66-2.90)  1  1.29 (0.71-2.33)  1.29 (0.60-2.79)  1  1.87 (0.91-3.80)  3.48 (1.64-7.37) | 0.17 |
|  | New York Heart Association functional class  I   - Robust - Pre-frail - Frail   II   - Robust - Pre-frail - Frail   III/IV   - Robust - Pre-frail - Frail | 1  1.80 (0.48-6.73)  4.78 (0.69-33.7)  1  1.34 (0.85-2.13)  2.46 (1.46-4.16)  1  1.01 (0.55-1.88)  1.26 (0.64-2.45) | 0.16 |
|  | Left ventricular ejection fraction  ≥40%   - Robust - Pre-frail - Frail   <40%   - Robust - Pre-frail - Frail | 1  1.51 (0.82-2.77)  2.17 (1.06-4.45)  1  1.08 (0.69-1.69)  1.86 (1.13-3.07) | 0.48 |
|  | No anemia   - Robust - Pre-frail - Frail   Anemia   - Robust - Pre-frail - Frail | 1  1.40 (0.93-2.12)  1.98 (1.20-3.26)  1  1.54 (0.79-3.00)  2.73 (1.34-5.56) | 0.70 |
|  | Serum creatinine below the median for the cohort (93umol/L)   - Robust - Pre-frail   Serum creatinine below the median for the cohort   - Robust - Pre-frail - Frail | 1  1.01 (0.58-1.74)  1.18 (0.63-2.21)  1  1.46 (0.93-2.28)  2.38 (1.44-3.95) | 0.78 |
|  | Not taking an ACE-I or angiotensin receptor blocker   - Robust - Pre-frail - Frail   Taking an ACE-I or angiotensin receptor blocker   - Robust - Pre-frail - Frail | 1  1.63 (0.89-2.97)  2.19 (1.10-4.34)  1  1.23 (0.80-1.89)  1.90 (1.17-3.09) | 0.49 |
|  | Not taking a beta-blocker  Taking a beta-blocker   - Robust - Pre-frail - Frail | NC  1  1.24 (0.85-1.80)  2.05 (1.35-3.12) | 0.31 |
|  | Body mass index ≤30kg/m^2^   - Robust - Pre-frail - Frail   Body mass index >30kg/m^2^   - Robust - Pre-frail - Frail | 1  1.34 (0.88-2.05)  1.95 (1.21-3.15)  1  1.27 (0.68-2.38)  1.85 (0.90-3.82) | 0.93 |
|  | MAGGIC risk score ≤14   - Robust - Pre-frail - Frail   MAGGIC risk score >14   - Robust - Pre-frail - Frail | 1  1.72 (0.96-3.08)  2.44 (1.21-4.89)  1  1.12 (0.72-1.74)  1.76 (1.08-2.85) | 0.44 |

**Supplementary Figure 1. Kaplan-Meier curves for mortality and heart failure (HF) hospitalization in women and men.** The sex-frailty interaction p-values were 0.25 for mortality and 0.44 for HF hospitalization.
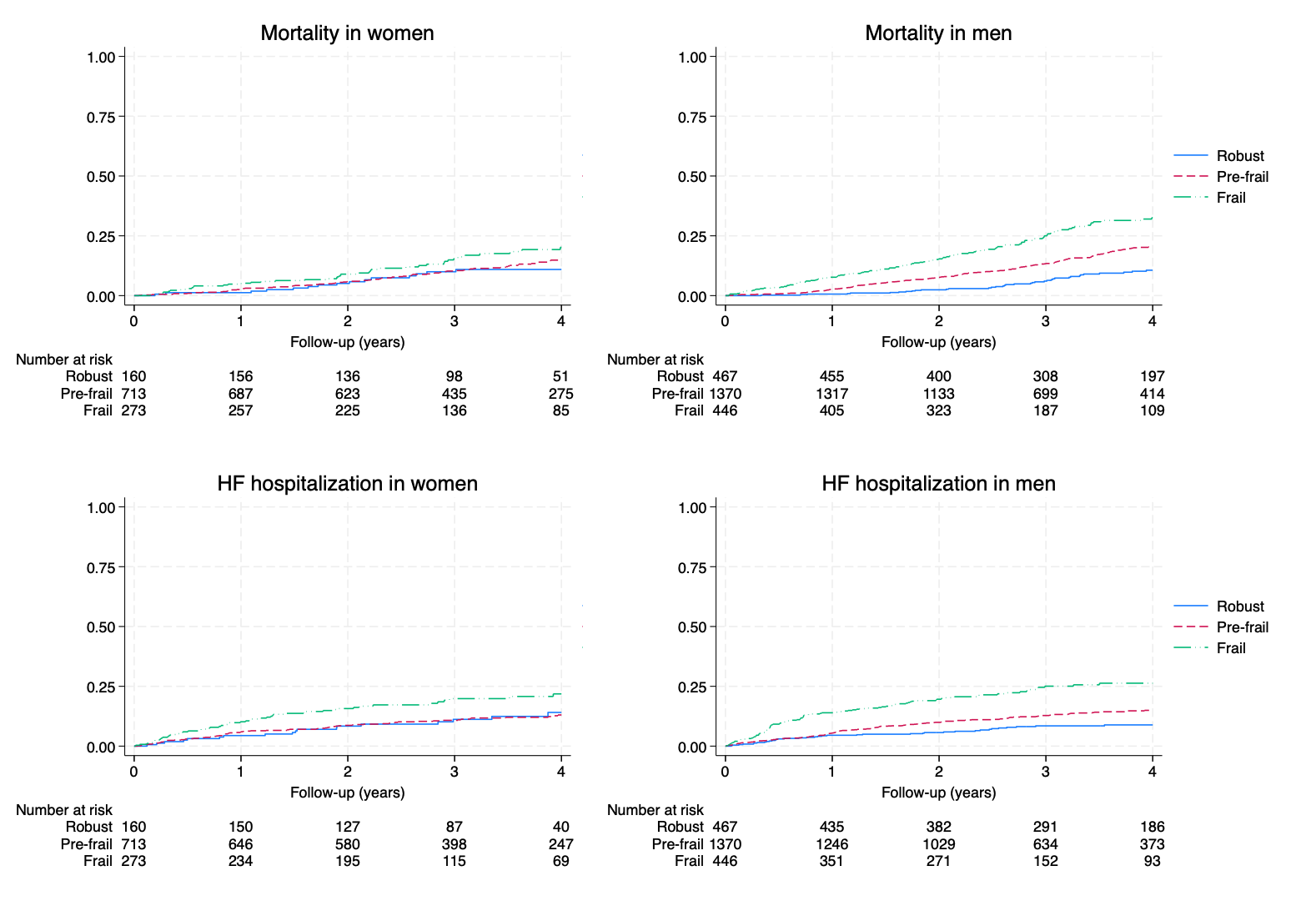


**Cumulative Deficit Index**

This index was created for each participant by assigning values of 0 or 1 depending on the absence or presence of each of the following: hypertension, hyperlipidemia, history of myocardial infarction, hospitalization within the previous two years, prior cardiac surgery, history of stroke, history venous thromboembolism, valvular heart disease, peripheral arterial disease, atrial fibrillation/flutter, chronic obstructive pulmonary disease, sleep apnea, chronic kidney disease, diabetes, HIV, history of tuberculosis, history of cancer requiring treatment, current smoking, anemia, body-mass index >30kg/m^2^, unintended weight loss, exhaustion. Deficits present for each participant were summed, yielding a score with a maximum possible value of 22, then each participant’s score was divided by 22 to calculate a deficit index with a minimum value of 0 and a maximum value of 1. We defined pre-frailty by cumulative deficit index as a cumulative deficit index >0.1 and ≤0.21, and frailty as a cumulative deficit index >0.21 as previously described(1).

**G-CHF Investigators**

**National Leaders (NLs), Principal Investigators (PIs), Co-Investigators (Co-Is), and Sub-Investigators (Sub-Is)**

***Study Principal Investigator:*** S. Yusuf

***Population Health Research Institute:*** P. Joseph, D. Leong, E. Lonn, M. Duong

***Argentina (552*):*** M. L. Diaz and J. M. Dominguez (National Leaders), S. Andrés Nemi, F. A. Bilancieri, S. Cardona, W. P. Casali, M. A. Castoldi, N. Cluigt, M. S. Copponi, G. Cursack, M. S. Cursack, R. G. Duran, C. F. Garcia, J. Hasbani, E. Hasbanl, M. A. Hominal, I. MacKinnon, P. E. Martinez Monferrán, A. Meirino, G. Paterlini, A. Picech, A. Prado, M. Rasmussen, D. G. Rojas, A. Rojas, V. L. Sernia, P. L. Varela, C. J. Zaidman, G. Zapata ***Botswana (229*):*** J. Mwita (National Leader), E. Botsile, M. Goepamang, C. Onen ***Brazil (686*):*** A. Avezum Jr. (National Leader), E. Abib Jr., A. S. Almeida, E. Azevedo, A. C. Barão, S. Z. Bazan, R. Bazan, L. Berreta, B. Biselli, N. Clausell, T. Colombo, O. M. Costa, V. De Barros, E. De Barros Correia, S. R. Y. de Campos, F. De Martino, L. J. G. Rodrigues de Paula, L. De Resende, R. De Sousa, A. S. B. De Souza, W. K. S. B. De Souza, G. De Souza Pantano, M. Del Monaco, D. Dumond, O. Dutra, E. Dytz, G. Fazolli, J. Ferreira, F. E. Giorgeto, P. G. Goldstein, R. B. Guimaraes, M. E. Hernandes, A. Humberto Vaz, S. Inuzuka, S. Jallad, A. A. Lacalle, M. A. Lemos, R. M. Lima, L. N. Maia, L. Massari, M. F. Mattiello, L. F. Medeiros, C. Minelli, M. A. Nakazone, F. Nobre, G. B. Oliveira, L. Oliveira, M. T. Oliveira, E. Ottz, R. Padilha, R. Pavanello, F. L. Peralva, W. Queiroz, G. Reis, E. Rejane Silva, B. Santana, N. L. Santos, T. L. Scudeler, M. Silva, A. Soeiro, A. Sousa, A. Souza, M. Teixeira, T. Torres, H. Vaz, H. Vilela, S. R. Youssef ***Cameroon (1365*):*** A. Dzudie (National Leader), C. K. Kouam, J. P. Abah, S. M. Abang, J. C. Ambassa, P. E. Anou, H. M. Ban'Haka, N. Clovis, S. Colette, S. A. N. Feuzeu, T. G. Fomekong, G. E. Manon, J. Ndjebet, N. Ngai, C. N. Nganou-Gnindjio, F. A. D. Ngongang, A. Nyanga, D. Palmer, N. Pierre, T. W. Sylvie, A. D. Tamdja, C. Tantchou ***Canada (1908*):*** C. Alba, A. Al-Hesayen, K. Anderson, M. Barrero, S. Bergeron, M. Bernier, F. Billia, C. Bourgault, B. Cantin, R. Costa, D. Delgado, A. J. Della Siega, A. Diaz, Y. P. Dubois, A. Ducharme, L. Duchesne, J. Ezekowitz, K. Flores, I. Gabizon, C.-E. Gagne, M. Garand, D. Hayami, G. Heckman, C. Henri, M.-H. Leblanc, C. Lemay, S. Lepage, E. Lonn, N. Lounsbury, M. McDonald, R. McKelvie, L. Mielniczuk G. Moe, J. Morin, G. Morgen, A. Munoz, S. Nelson, K. O’Connor, E. O'Meara, Y. Pépin Dubois, G. Proulx, N. Racine, M. Rajda, A. Raymond, P. Rheault, H. Ross, J. Rouleau, A. Schaffer, M. Senechal, Y. T. Sia, R. Singh, S. Smith, E. Swiggum, M. Toma, F. Tournoux, M. White, A. Yip, S. Zieroth ***Chile (359*):*** F. Lanas (National Leader), B. Bobadilla, G. Jano, M. Medina, S. Saavedra, M. Vega ***China (1764*):*** J. Zhu and H. Tan (National Leaders), F. Ai, X. Bai, X. Chen, Y. Chen, X. Gao, J. W. Gou, X. Hao, Z. Jia, H. Jiang, W. Jin, T. Liang, X. W. Liang, Y. Liang, H. Y. Luo, J. Peng, S. Qin, L. Ruihong, H. Tan, G. Tan, X. Wang, T. Wang, M. Wang, F. Wei, J. Wu, J. Xu, J. Yanhong, X. Yang, W. Yuanyuan, X. Yun, T. Zheng, W. Zhijun, W. Zhou ***Colombia (807*):*** J. Gomez-Mesa (National Leader), T. Alvarez, W. B. Ariza, A. M. Baron, M. A. Betanar Diaz, S. Bermudez, B. E. R. Blanco, J. Buelvas W. A. Buelvas Argumedo, A. Buitrago, A. Cadena Bonfanti, H. Calvo, E. M. M. Carreno, L. P. Castano, E. Conmenos, L. A. Caycedo, J. A. Ceron, F. A. T. Dada, V. De La Esprula, N. A. Florez, D. I. M. De Salazar, S. Del Pilar Baracaldo Gimet, J. E. V. Eljach, S. Galindo, A. Garcia, F. Gomez, H. G. Lozada, A. Marino, E. Martinez, G. A. Martinez, J. L.. A. Mendoza, A. P. F. Monten, M. C. Montes, A. P. F. Monton, G. S. Moreno, N. Murillo, P. Olaya, M. M. D. Olite, S. Pardo, J. D. L. Ponce de Leon, S. Redondo, V. A. Reyes, C. E. Rivera, N. Rodríguez, M. Rojas, J. Rueda, J. F. Salazar, J. Sandoval, F. A. Solano, J. M. Torres, J. M. Valiente, J. E. Vengas Eljach, V. Zolgava***Denmark (268*):*** K. Kragholm (National Leader), G. Gislason, S. H. Jørgensen, G. Nielsen, M. W. Pedersen, P. V. Rasmussen, P. Søgaard, H. Wiggers ***Ecuador (498*):*** P. Lopez-Jaramillo (National Leader), N. Anzules, S. Caceres, S. Carrasco, W. Duarte, Y. C. Duarte, I. Herrera, M. S. Jaramillo, D. Jimenez, P. Llomes, F. P. C. Long, J. Lopez, E. Paez, P. Perez, F. Ponce, B. Saenz, F. Trujillo, E. Vargas, M. S. Velez, M. L. Villota, I. Zuelta ***Egypt (230*):*** K. Wagdy Shaker (National Leader), P. P. Selwanos, M. Yacoub ***France (162*):*** F. Alla (National Leader), R. Boulestreav, N. Bourrelly, N. Delarche, A. Didier, L.-L. Diene, F. Federica, P. Gosse, S. Goussot, R. Gueche, J. Paul Guillot, K. H. Hamou, F. Jourda, D. Kenizou, M. Mansour, G. Papaïoannou, E. Rugina, F. X. Soto, I.-M. Suzenne, J. Michel Tartiere ***Germany (1300*):*** G. Ertl (National Leader), T. Wittlinger (National Leader), P. Alger, I. Alsoudi, S.-E. Baller, J. Becher, E. Blank, M. Bockmann, K. Boelmans, M. C Bott, F. Brattinger, S. Brenner, M. Breunig, O. Bruder, A. Carstguson, V. Cejka, M. Christa, E. Cramer, M. Drexler, J. Friedenberger, P. Goldstein, R. Guimaraes, A. Hagenow, A. Hauber, D. Hausmann, K. Hertting, R. Hintze, W. Ito, A. Jabs, M. Junge, W. Jungmair, C. Kadel, Z. Kiraly, S. Kohler, B. Kraus, S. Loebis, B. Lutsyuk, H. Mahrholdt, S. Maier, H. Martin, G. Mentz, J. McChord, H. Mollinger, C. Morbach, Z. Moussaoui, C. Muller, H. Nagele, I. Neufeld, S. Neugebauer, P. Ons, K. Oppenlaender, L. Peters, C. Potolidis, W. Raut, W. Rieker, A. Rieser, S. Schellong, R. Schueler, I. Seifert, M. Specking, M. Speth-Nitschke, F. Stahl, A. Stief, D. Stierle, T. Stoerk, S. Stork, T. Twisselmann, I. Voigt, B. Voigts, M. Weisbach, C. Weiss, S. Wilke, C. Widhalm, A. Wolf, M. Wurdak, M. Zaczkiewicz, O. Zimmermann ***India (2212*):*** A. Roy (National Leader), J. Abdullakutty, S. Asotra, S. Chandra, H. Chaturvedi, G. Chaudley, D. Desai, J. Georgi, R. Gupta, N. Jathappa, D. Kamath, S. Karna, K. P Kumar, B. H. Lokesh, J. S. Makkar, B. Mohan, N. Naik, P. Negi, M. P. Purayil, R. S. Rao, S. Seth, G. Sharma, B. Singh, S. Singh, M. A. Srilakshmi, R. Tandon, A. Tewari, K. Varghese, S. K. Verma, D. Xavier, R. Yadav ***Italy and Switzerland (577*):*** A. Maggioni (National Leader), E. Capati, A. Cecchi, A. Cherubini, M. Concilio, F. Cosmi, C. Crljenica, S. D'Orazio, G. M. Francese, A. Frisinghelli, M. Gnemmi, M. Gulizia, C. Marcassa, T. Moccetti, C. Monti, R. Nuti, F. Orso, M. Palvarini, E. Pasotti, G. Piccinni, M. Piepoli, M. Rossi, G. Russo, A. Salvicchi, F. R. Scopigni, P. Temporelli, F. Venturi, A. Verde, G. Q Villani ***Kenya (290*):*** G. Yonga (National Leader), F. Barasa, E. Mbugua, W. Nalwa ***Mexico (51*):*** J. A. Magaña Serrano (National Leader), R. K. N. Carrazco, E. Castro-Montes, P. G. D. Flores, J. A. C Lopez, M. Silva, M. Vazquez ***Mozambique (146*):*** A. Damasceno (National Leader) ***Nepal (342*):*** S. Sharma (National Leader), Y. Bhatta, N. Bhurtyal, Sa. Gautam, Sw. Gautam, T. Y. Ghising, V. Kattel, D. R. Mishra, A. Rai, R. Rana, R. Sapkota, D. Sharma, N. Shrestha, S. L. Shrestha, M. Subedi, R. Tamrakar, P. M. Tripathi ***Nigeria (1290*):*** K. Karaye (National Leader), H. Abubakar, V. Ansa, N. Ishaq, A. Kabir, O. Ogah, H. Saidu ***Pakistan (421*):*** K. Kazmi (National Leader), B. Ahmed, A. Artani, A. M. Kayani, A. Laghari, R. Munir ***Philippines (130*):*** A. Dans (National Leader), J. Cruz, L. K. Evangelista, M. Mercado, D. Morales, D. F. T. Morales, D. Sulit, L. Tirador, C. Trompeta ***Poland (708*):*** A. Budaj (National Leader), S. Anna, U. Baburz, E. Bakuka, J. Bednarski, A. Cheudoba, A. Chmielinski, M. Ciuraj-Hanczarek, K. Cymerman, A. Czepiel, T. Czerski, D. Dabrowski, J. Gebalska, M. Gmytrasiewicz, J. Gniot, M. Grelak, U. Grochowicz, T. Imiela, M. Korol, C. Lewandowski, B. Magdalena, A. Maicka, U. Matys, B. Mierzejewska, B. Miklaszewicz, E. Mirek-Bryniarska, A. Nowak, M. Owsiak, Ł. Pastwa, J. Patryn, E. Piechocka, M. Piepiorka, A. Pracka, A. Redmarika, R. Sicursa, M. Skorski, M. Solkiewicz, A. Stasiwski, E. Struzik, W. Sudnik, A. Swiderska, M. Wujkowski, J. Ziolo ***Portugal (145*):*** J. Silva Cardoso (National Leader), S. Amorim, A. Andrade, P. Araujo, A. Baptista, J. Cabral, M. Campelo, A. Cavalheiro, C. Ferriera, H. Guedes, A. R. Godinho, L. Marques, A. Meto, E. Moreira, I. Moreira, B. Moura, I. Oliveira, R. Pinto, R. Samtos, D. Seabra, P. Silva, A. Sousa, H. Vasconcelos***Russia (602*):*** N. Pogosova (National Leader), S. Isakova, S. Ivan, N. Kovalkova, D. Panov, A. Salbieva, Z. Sergey, Y. Yufereva, A. Yusubova, V. Gafarov, I. Osipova, T. Poponina, Y. Pozdnyakov, Z. Sizova ***Saudi Arabia (903*):*** K. Alhabib (National Leader), S. Alasmari, K. Alghalayini, M. Elreadaisy, W. A. Habeeb, Y. Kassim, A. Kinsara, S. Lawand, S. Monem, U. Ullah Jan, M. Zeyad ***South Africa (111*):*** K. Sliwa (National Leader), M. De Andrade, J. Fortein, E. M. Makotoko, K. Moeketsi, P. A. Moses, P. Mntla, M. J. Mphahlele, M. Ntsekhe, N. T. Poswa, A. Ratsela, B. M. Thomas, K. M. Thomas, C. Viljoen, A. Xana, Y. S. I. N. Yiga ***Spain (572*):*** A. Bayes-Genis (National Leader), L. C. Belarte Tornero, S. R. Bustillo, E. B. Caballero, J. C. Castillo, M. G. Crespo-Leiro, R. De La Espriella, N . Farre, P. Garcia-Pavia, I. P. Garrido Bravo, J. Lopez, D. Mallon, A. Manuel, M. C. Marcos, G. Minana, M. J. Paniagua Martin, D. A. Pascual-Figal, J. Nunez, F. Perez, J. L. Roses, E. Santiago, I. Zegri Reiriz, ***Sudan (504*):*** A. ElSayed (National Leader) ***Sweden and Iceland (486*):*** L. Lund (National Leader), S. Bandh, K. Boman, N. Z Floderer, M. Fu, V. Goloskokova, C. Hage, C.-J. Lindholm, J. Lindström, I. Lönnberg, E. Marosi, D. Nurring, A. Rafnsson, J. Sandstrom, B. Szabo, T. Thorvaldsen, J. Thulin, K. Vargova ***Tanzania (778*):*** A. Makubi (National Leader), B. Alphonce, E. Alphonce, P. Chillo, R. Fabiano, M. Janabi, F. Kalokola, S. Kanenda, J. Kataraihya, P. Kisenge, J. R. Meda, R. Mutagayma, B. Mwakalukwa, R. Vogt, H. Vogt ***Turkey (493*):*** A. Temizhan (National Leader), D. R. Acar, Y. Çavuşoğlu, A. Celik, H. E. O. Cetin, A. A. Erenoglu, S. Gulec, N. Ozyuncu, M. B. Yilmaz ***Uganda (644*):*** C. Mondo (National Leader), P. Ingabrie, C. Lugero, Z. W. Zhu, ***Ukraine (505*):*** A. Parkhomenko (National Leader), S. Andriyevska, L. Chernachuk, S. Fedorov, E. Filatova, N. Lipkan, K. Lobanova, Sh. Oleksandr, Sk. Oleksandr, V. Pidlisna, V. Plohotnyk, S. Shvaykin, K. Valentyna, O. Verbovska, L. Vereschuk, L. Voronkov, Y. Zalisna, Y. Zalizna, N. Zenchenco ***United Arab Emirates (198*):*** A. Almulla (National Leader), F. Bader, N. Bazargani, M. Elbanna, G. Gabra, I. Hamour, M. Khalil, B. Mohamed, J. K. Praveen, H. Sabbour, M. Soliman, ***United Kingdom and Ireland (322*):*** M. O'Donnell (National Leader), C. Arden, J. Barton, P. Donnelly, T. Edwards, J. Foster, A. Fuat, A. Jackson, Z. Jan, P. Jhund, T. Kiernan, B. Mcadam, N. McAleavy, A. Moriarty, A. Murphy, C. Orr, F. Witherow ***United States of America (783*):*** K. Branch and J. Probstfield (National Leaders), S. Adams, F. Adler, S. Airhart, M. Aldridge, R. Arimie, M. Arnold, D. Brautigam, K. Breathett, N. Christian, A. Desai, M. Friedman, H. Gaggin, C. Galvin, R. Gopalan, T. Gorman, M. Gulati, M. Hutchinson, N. Ibrahim, M. Jones, E. Juneman, T. Kitchen, W. J. Kostis, W. Kostis, K. Lotun, V. Malhotra, K. Maurice, R. Mehta, M. A. Meier, M. Meirer, A. E. Moreyra, S. Murthy, R. V. Patak, I. Piña, G. Reeves, R. Shetty, N. K. Sweitzer, J. Tauras, S. Thew, J. Vivian, M. Wagmeister, S. Westfall, D. Whellan

**References**

1. Farooqi MAM, Gerstein H, Yusuf S, Leong DP. Accumulation of Deficits as a Key Risk Factor for Cardiovascular Morbidity and Mortality: A Pooled Analysis of 154 000 Individuals. J Am Heart Assoc. 2020;9(3):e014686.
